# A fragile and divided European Union meets Covid-19: further disintegration or ‘Hamiltonian moment’?

**DOI:** 10.1007/s40812-020-00165-8

**Published:** 2020-07-17

**Authors:** Giuseppe Celi, Dario Guarascio, Annamaria Simonazzi

**Affiliations:** 1 University of Foggia, Foggia, Italy; 2 Sapienza University of Rome, Rome, Italy

**Keywords:** Core-periphery relations, Euro crisis, EMU, Global value chains, F02, F15, F45, F55

## Abstract

Despite being symmetric in its very nature, the Covid-19 shock is affecting European economies in a very asymmetric way, threatening to deepen the divide between core and peripheral countries even more. It is not Covid-19 itself, however, but the contradictions within the EU’s growth model and institutional architecture that would be to blame for such an outcome. The dramatic impact of the economic crisis brought on by the pandemic and the threat that it poses to Eurozone survival seem to have forced a reluctant Germany into action: a minor step, but an important signal. This note analyses the crossroads currently facing Europe—the risk of disintegration vis-a-vis the opportunity for a ‘Hamiltonian moment’—discussing possible future scenarios in the light of past developments.

## Introduction

Like viruses, crises too can rapidly change their DNA: the financial crisis of 2008 changed from international to regional, from financial to real, eventually turning into an existential threat to the whole European integration project. In the institutional context of the Eurozone (EZ), the financial crisis soon developed into a sovereign debt crisis, dragging the banks along with it. In the austerity environment that followed, the southern periphery (SP) never completely recovered the losses in output, employment, and fiscal sustainability. Thus, the “symmetric” coronavirus shock hit countries that were in highly asymmetric conditions. In fact, not all the countries of the Union have the resources needed to intervene in support of their economy, prompting concern that countries with the deepest pockets might be getting an unfair advantage in the EU’s single market. Far from triggering mutual protection, the Covid-19 crisis seems to be paving the way for the same mistakes that followed the 2008 financial crisis. The centrifugal forces threatening disintegration of the European Monetary Union (EMU) seem to have been defused, albeit only in part and only in extremis, at least for the time being. However, the survival of the Union depends not only on responding to the severe financial problems caused by the epidemic, but also means addressing the long-term, structural problems that led to the increasing divergences among her members. As Chancellor Merkel herself acknowledged, “It is in nobody’s interest for Germany alone to be strong after the crisis”.^1^ Convergence is essential to put the Union on a more solid basis so as to guarantee its long-term sustainability.

What policies and what reforms should be implemented to pursue this objective? And are they economically and politically feasible? Trying to answer these questions, we shall briefly review the institutional and structural causes of the increasing divergence between core and SP, shedding light on three momentous events: the creation of the monetary union, the 2008 financial crisis and the Covid-19 shock.

## A brief history of EU divergence in three steps

### Before the 2008 crisis

The first decade following the introduction of the EMU saw continuity in the process of Europeanisation embarked upon as from the formation of the Common Market, based on financial liberalization and market globalization. As argued in Celi et al. (2018,2019), Europeanisation meant EU-wide application of a policy of deregulation of goods, labour and capital markets that affected the timing, shape and direction of the European integration process, halting the process of convergence between the core and the SP of the EU. The more developed core (centred on Germany) increased its productive and technological capacity; the SP, caught between product competition within the EU and cost competition from emerging economies in the international markets, saw a decline in its manufacturing capacity.^2^

With the fall of the Soviet Union and the entry of the former Socialist countries of Central and Eastern Europe in the EU, the Eastern Periphery (EP) became a key gear of Germany’s manufacturing matrix (Stehrer and Stollinger 2015). A huge flow of direct investments, primarily in the automotive sector, transformed the economies of the Visegrad Pact (Poland, Hungry, Slovakia, and Check Republic) into an essential source of intermediate goods (medium and medium–high quality) for the German industry. A well-qualified, extremely cheap workforce, generous subsidies and tax breaks, as well as geographical proximity and historical links, are among the determining factors of the increasingly tight links between the core and its EP.

The impressive growth in manufacturing capacity in the East led to a restructuring in the hierarchical organization of the supply chains across Europe: the weaker suppliers in the South were displaced by their cheaper competitors in the East, while the highly specialised suppliers of components in the industrial regions of the South maintained, and even increased, their close links with the German producers.^3^ The crowding-out of the less dynamic firms in the SP did not take the form of efficiency-enhancing market selection but rather a generalized reduction of production capacity, contributing to fuel a well-documented (see, among the others, Guarascio and Simonazzi 2016; Dosi et al. 2019) process of ‘poor tertiarisation’ of the SP. On the other hand, the EP’s industrial miracle was created by foreign, mostly German, direct investment, with the automotive sector taking the lion’s share. So far, we have seen no comparable development of other productive sectors, nor has the automotive sector created spill-over effects in the rest of the economy (Krzywdzinski 2019). On the contrary, the surge in the production of components for the automotive sector has partly displaced other productions, leading to an increasing ‘mono-specialization’ of these economies. Despite a growing shortage of skilled labour, wages have remained modest. Threats of production shifting further East, to Romania, Turkey, or to North Africa, (Pavlinek et al. 2017)^4^ are reflected in the adoption of a wage containment policy at home, driving young people with high educational qualifications to emigrate, and weakening the countries’ skills base. With domestic demand subdued, the high growth rates recorded by these countries are entirely led by the growth in exports of local production by foreign multinationals (i.e., the so-called “integrated peripheral markets”). While their intensive specialisation in the automotive industry makes them totally dependent on the health of the German automotive industry, the foreign control of production decisions, innovation processes and markets makes it extremely difficult to undertake an independent, less unbalanced development path (Celi et al. 2018).

To conclude, the two peripheries—the Southern one, made up of the Mediterranean economies, and the Eastern one, with the prominent role of the Visegrad countries—suffer from different fragilities, which descend from their common, albeit diverse, economic and financial dependence on the core. However, the core itself is dependent for its growth on the pattern of specialisation within the EU: the Southern markets providing an outlet for its increasing surplus of manufactures, the Eastern countries supplying cheap inputs for its industries. This combination of structural divergence and economic interdependence lies behind the fragility of the Union as well as of the improbability of its disintegration given the high costs it would entail for core and peripheries alike.

### The age of austerity

In the first period of the EMU (2000–2008), the core-SP structural divergence was partly hidden by massive financial flows to the periphery. The 2008 financial crisis, and the ensuing international liquidity crunch, prompted a “sudden stop” of capital flows and a collapse in demand and imports. At that point, the structural and institutional flaws of the EMU became evident: the reaction to the crisis aggravated the divergence. With the blame for the crisis put squarely on borrowers, austerity policies were advocated (or imposed) to ensure debtor countries’ public and private solvency.

With austerity killing demand, growth and imports in the SP, Germany, which had built most of its huge trade surplus between 2003 and 2008 by exporting to the periphery, had to find new outlets for its goods. Special international conditions—namely, China’s huge growth, which gobbled up German capital goods and high-quality durable consumer products (particularly cars), and the vigorous American recovery—supported Germany’s ability to redirect its trade flows, expand its market shares outside the EMU, and make a speedy return to its pre-crisis production levels. The United Kingdom, the United States, but above all China, became the most important markets for German exports. The rapid recovery of the German economy pulled the EP along with it: the Visegrad countries recorded unparalleled growth in Europe.

**Fig. 1:**
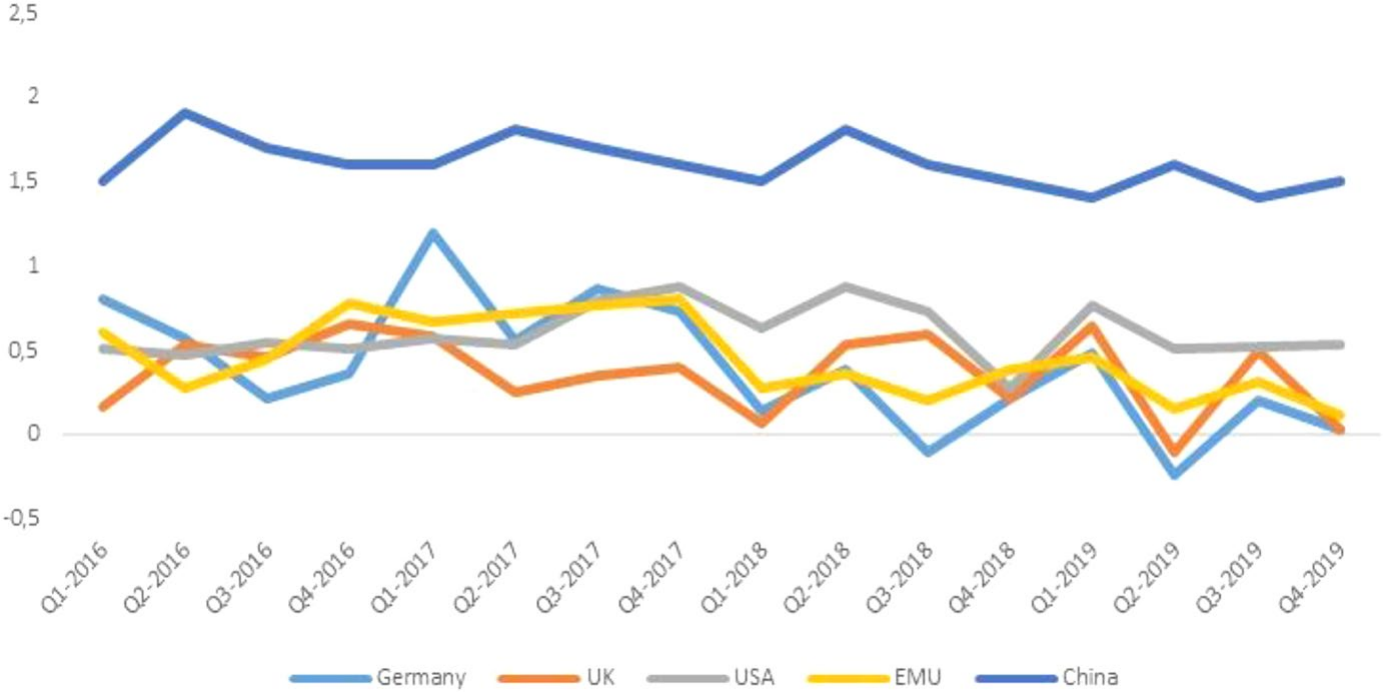
GDP per capita (rate of change over the previous quarter, 2016–2019). Source: authors’ elaboration on OECD data

With the abrupt change in the international scenario in 2016, Germany’s (and the entire EMU’s) mercantilist strategy was up against the ropes. The Brexit referendum, Trump’s election, and the U-turn in Chinese economic policy inaugurated a phase of retreat in international trade. Trade with the UK began to suffer due to the increasing uncertainty in future trade relations. When the United States took action to reduce the external deficit, China and Germany, the countries with the largest trade surpluses vis-à-vis the United States, were caught in the crosshairs. Trade tensions between the US and China put further pressure on international trade. The export-led growth model that had so far supported Germany’s leadership began to creak. The change in world trade took its toll on German (and EU) growth rates. From the second quarter of 2017, the slowdown in German exports hit industrial production and the GDP, widening the growth gap with China and the USA and dragging the whole EMU along with it (Fig. 1).

As the escalation of trade disputes affected relations between the United States and Germany^5^ (and by extension the EU), the negative effects on Europe’s (export-led) growth intensified. In the last quarter of 2019, just a few months before the outbreak of Covid-19 in the EU, Germany’s growth rate zeroed. Income growth estimates for the rest of Europe were consequently reduced.

### Enters the Covid-19

**Fig. 2:**
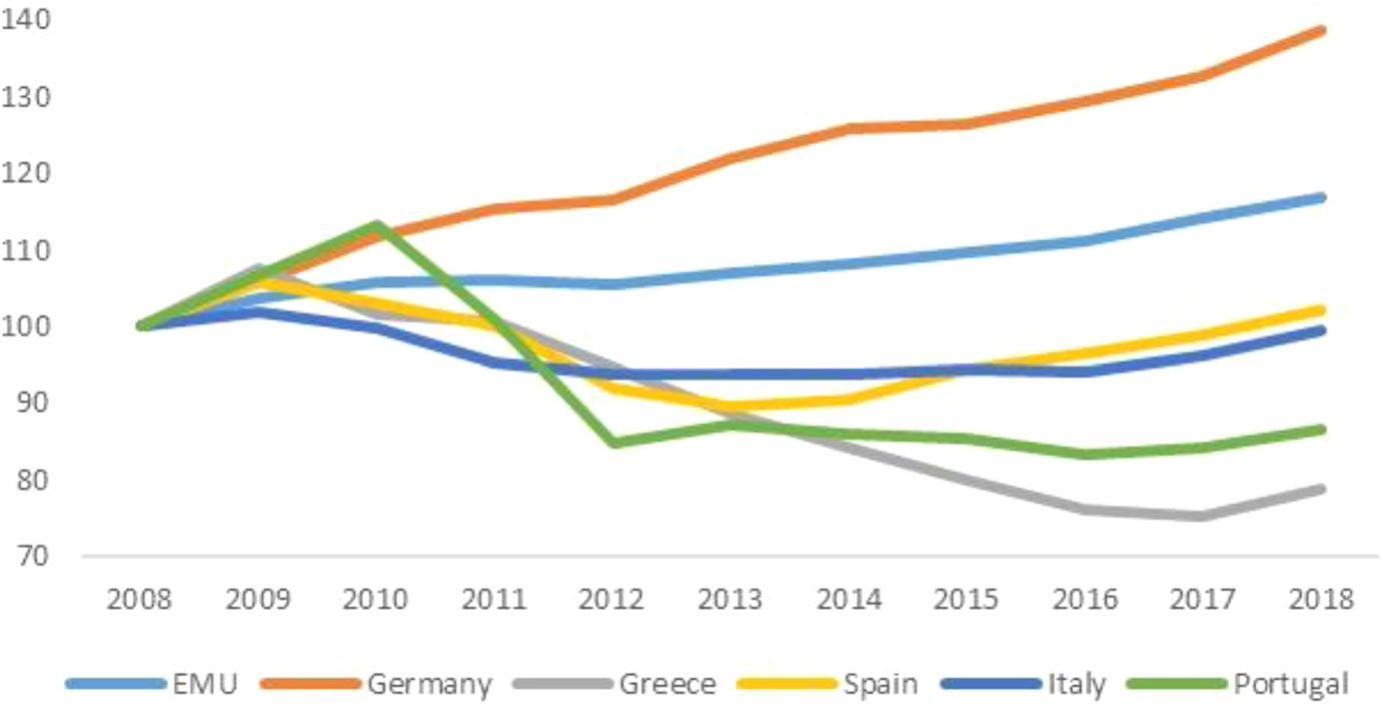
Public spending on education. EMU, Germany and SP's countries. Source: Authors’ elaboration on Eurostat data

**Fig. 3:**
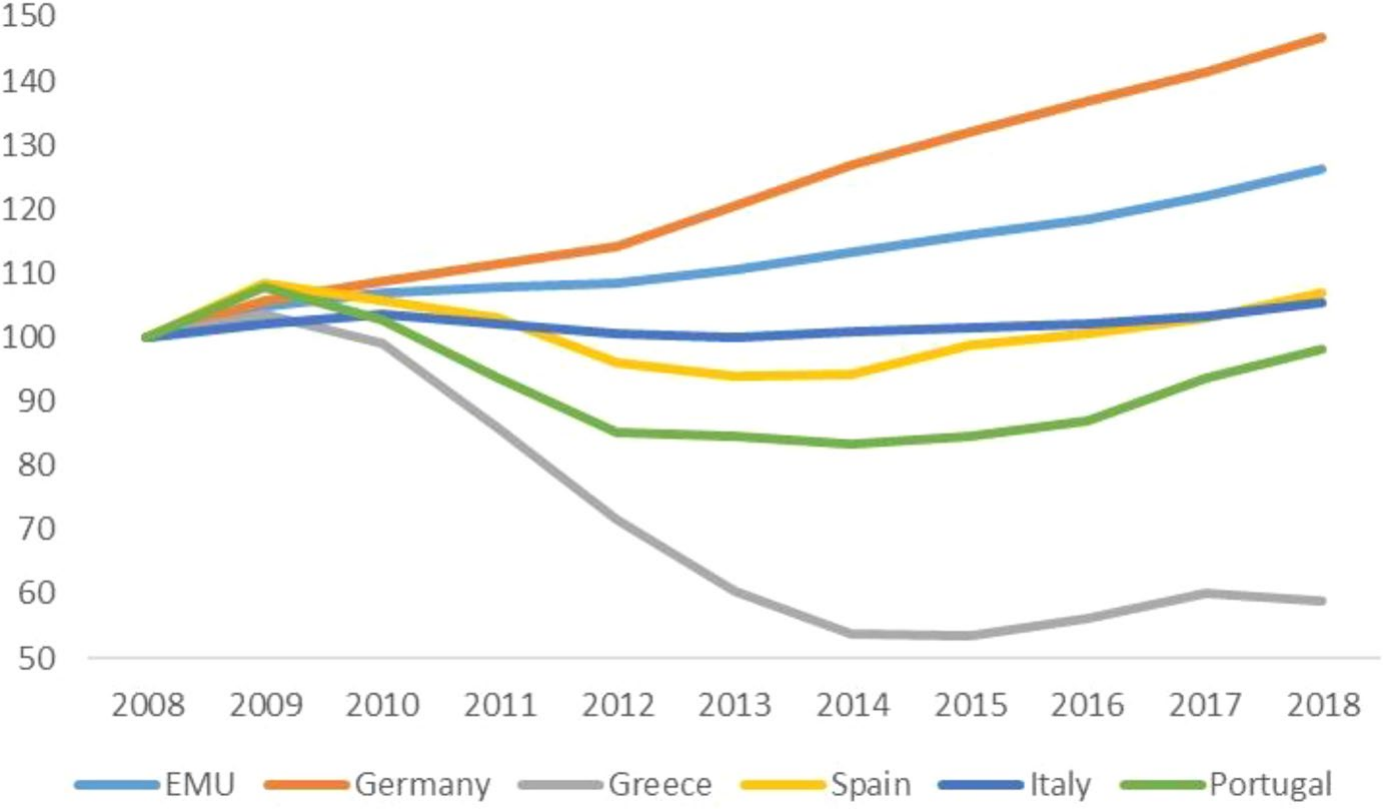
Public spending on health (general). EMU, Germany and SP's countries. Source: authors’ elaboration on Eurostat data

**Fig. 4:**
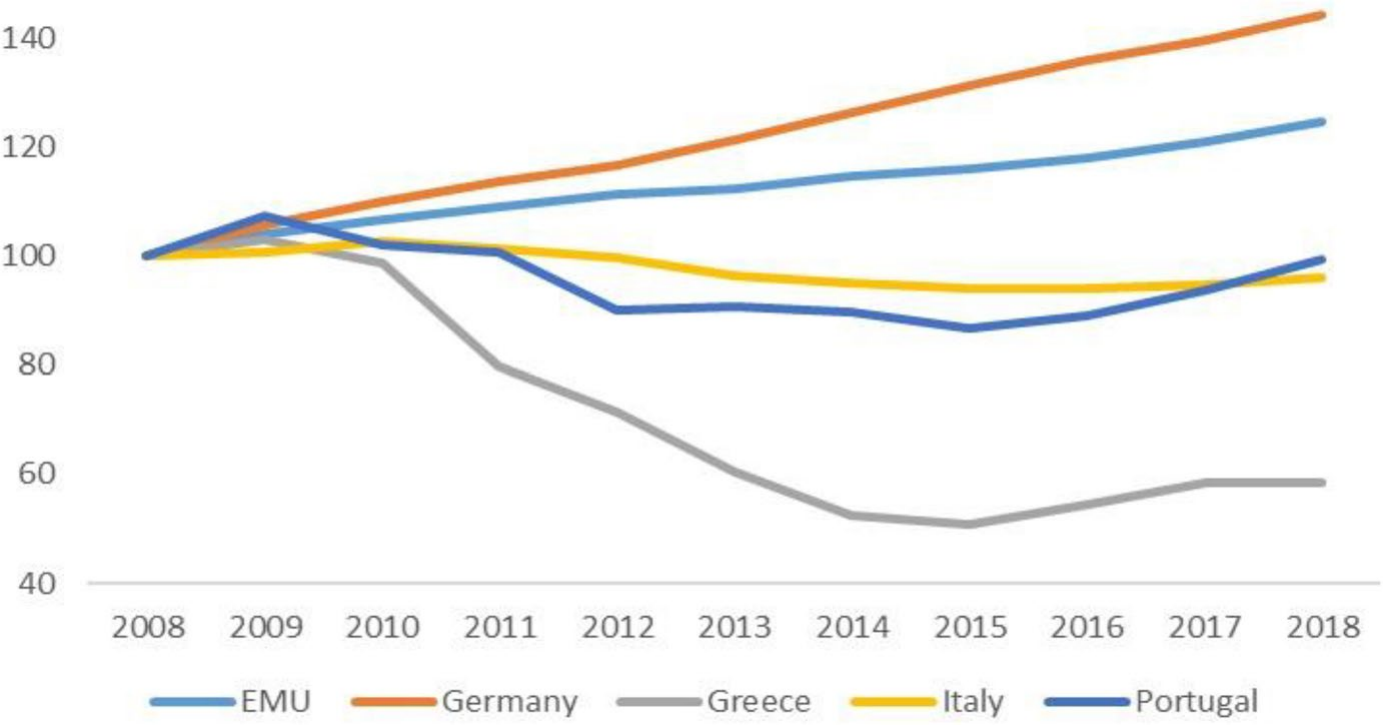
Public spending on health (hospitals). EMU, Germany and selection of SP’s countries. Source: authors’ elaboration on Eurostat data

The pandemic arrived in Europe from the south: Italy was the first country to suffer the contagion. Its abrupt, dramatic effects exposed the fragility of the periphery and the crippling effects of austerity policies. Since 2010, across the board cuts in social spending had hit the entire range, from health to education, from social assistance to social investment.^6^ Figures 2, 3 and 4 show the evolution of the share of public expenditure on education and health (divided between general expenditure and hospitals) relative to GDP in the EMU, Germany and the SP between 2008 and 2018. Many hospitals had been closed, the number of beds reduced, medical and nursing staff cut back (for a detailed analysis of the impact that austerity policies had on the Italian health care system, see Prante et al. 2020). It is not surprising that the death toll was higher where intensive care facilities were scarcer. On the eve of the Covid crisis, public health accounted for 6.5 percent of the social product in Italy and Spain, and almost 10% in Germany, where per capita healthcare spending did not suffer cuts due to austerity (though it was not completely spared self-imposed restrictions). The Covid-19 exposed another aspect of the ‘divisive’ Union (Celi et al. 2020): different capacities to respond to the pandemic crisis.

Economic ideology shares with austerity the responsibility for the scant endowment of medical equipment and health staff. Efficiency, understood as cost reduction, has been taken as the guiding principle. The obsession with competitiveness and reliance solely on the export-led growth model accounts for the almost exclusive emphasis on “tradable” sectors, to the detriment of “non-tradable” sectors (housing, health, education, welfare services in general), considered of lesser importance for international competition. This means that, in the era of austerity, these items have been the first to be sacrificed, in debtor and creditor countries alike. Chazan (2020) reports that for years, politicians and health economists in Germany have complained that the country has too many hospitals, with the Bertelsmann Foundation recommending halving the number of hospital, from 1400 to fewer than 600 (Chazan 2020). Only such a radical consolidation—the Bertelsmann study argued—would “improve patient care and mitigate the shortage of doctors and nursing staff”. The pandemic succeeded in transforming this “oversupply” into an asset.

The same logic of pursuing the lowest cost guided the international location of production, which displaced domestic production and weakened production capacity in the SP. From a regional (European) point of view, this process resulted in a reorganisation of production and trade relations between core, EP and SP. On a global scale, core and peripheries entered into very long and complex GVCs that proved extremely vulnerable in the face of the interruptions prompted by the pandemic. Personal protective equipment, respirators, medicines: the emergency has made it clear what it means to lose the capacity to produce domestically, both in quantity and quality, what is urgently needed, bringing the problem of self-sufficiency back to the attention of economists and policymakers.

## Long-term consequences of short-sighted responses

### Symmetric shock, asymmetric consequences

**Fig. 5:**
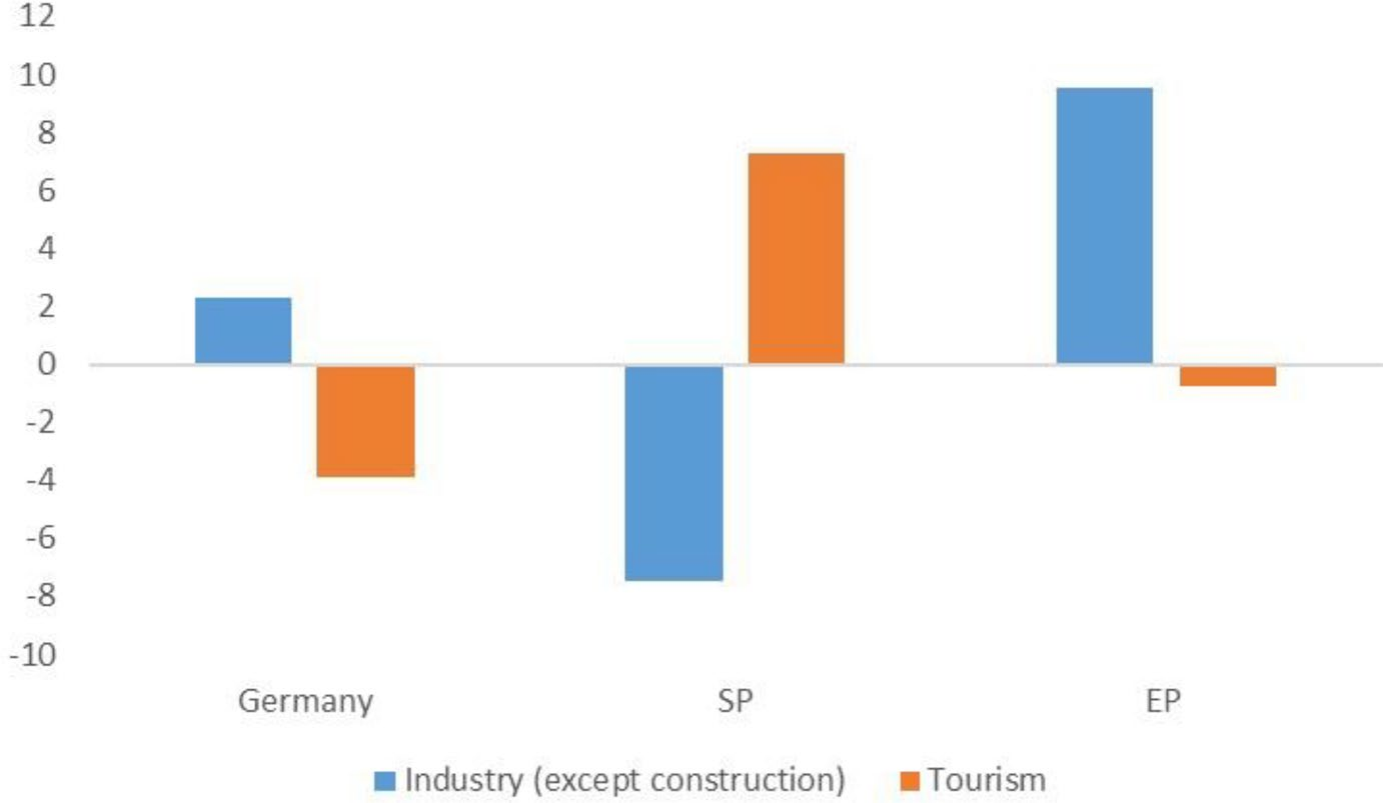
Number of active enterprises: industry (except construction) and tourism. Rate of change (2010–2017), Germany, SP and EP. Source: authors’ elaboration on Eurostat data. Note: Tourism includes restaurants, accommodation and travel operators

There is no such thing as a symmetric shock. In addition to the grim toll of victims and the incredible pressure on the health systems of all countries, the lockdown of activities to reduce contagion meant a tremendous plunge in production and incomes and enormous pressure on public finances all over the world. However, the lockdown is expected to affect economies differently. The Central and Eastern European countries have been less affected by Covid than the Western European countries: not trusting the resilience of their fragile health systems, they have had to rely on rigid social distancing (Walker and Smith 2020). Even within this group of countries there are differences: thanks to their more robust health systems, the Czech Republic and Slovenia were less constrained by rigid social distancing and able to start economic recovery earlier. Moreover, due to their strong productive links with Austria—a country relatively less affected by the pandemic which came out of the lockdown earlier—and their favourable positioning in the development of digital economy (Wiiw 2020), their economic outlook is rather better. Conversely, it will be tougher for the economies, like those of the SP, which are more dependent on services—tourism and hospitality in particular (Fig. 5)—and for CEE countries and southern regions that rely to a greater extent on production of intermediate products for final producers, since the latter can better defend themselves from fall in demand by cutting down orders to their suppliers (the so-called “whip effect”).

Policies have also differed widely across countries and regions. While all the central banks of the developed world promptly intervened to provide almost unlimited liquidity, in the EU public spending in support of the economy has been left to the national states (on this point, see the contributions to this Forum by Landesmann and Heimberger et al.). Although the Stability Pact has been temporarily suspended,^7^ there are obvious differences in how much member states can spend, depending on their fiscal space. Member states are making use of the new flexibility granted by the EC on state aid rules, strictly enforced beforehand to ensure fair competition within the internal market (Rios 2020). Germany, which accounts for about a quarter of the EU’s GDP, accounts for more than half (52%) of the emergency coronavirus state aid approved by the EC, prompting concerns that countries with the deepest pockets might be getting an unfair advantage by such a sudden (and temporary) abandonment of one of the Common Market’s key pillar (France and Italy each account for 17% of the total). An EU official, speaking on condition of anonymity, observed that “if you look at the scale of what Germany in particular, but also some others, are doing—any notion of level playing field or single market integrity has gone out of the window.”^8^

### Debt sustainability: public, private

These concerns underpin the ailing south’s demand for a joint EU financial plan. In the absence of a prompt and massive common effort, the SP will pay the highest price to the health crisis. Indeed, the different firepower will entail a still greater asymmetry in the economic and power relations between the various member states.

The ECB, alone among the Eurozone institutions, is doing as much as it can to avoid breakdown of the EMU. To address the Covid-19 crisis, it launched a new asset purchasing programme: the Eurosystem’s balance sheet shot up from 4692 billion Euros on 28 February to 5395 billion by 1st May 2020. Despite this massive monetary injection (700 billion in two months) the spread on Italian bonds, which had fallen in mid-March following the ECB’s announcements, again rose very rapidly, fluctuating in response to political developments. Indeed, as Tooze and Schularick (2020) point out, if, in the 2008 crisis, the liquidity injected into the system by the ECB was enough to prevent deflagration of the banking system,^9^ the current crisis would require a coordinated fiscal policy of enormous proportions. Despite some recent moves (inaugurated by a Merkel–Macron agreement), this still does not seem to be looming on the horizon. The newly released ‘Next Generation’ (NG) plan, based on the 2021–2027 budget, celebrated by some as a “Hamiltonian moment”, has yet to qualify as forerunner of an EU-wide up-to-the–challenge fiscal capacity.^10^ First of all, it is meant to be temporary and, moreover, it is too little, too late. The Plan should mobilize 750 billion euros, 500 in the form of grants and 250 in loans. Apart from the fact that these are gross figures—once the member states’ contributions to the EU budget are subtracted, the net amount received by the neediest countries is much smaller—their disbursement will not start before 2021, will be distributed over a 4-year period, with amounts that grow over time, and, as stated in the EC’s “Proposal for a Regulation” the financial contribution will “be paid in instalments once the Member State has satisfactorily implemented the relevant milestones and targets identified in relation to the implementation of the recovery and resilience plan” (EC 2020, art. 17.4.a). As Darvas (2020) emphasizes, the incorporation of the NG plan into the EU’s next multiannual budget would take advantage of a well-established framework, ‘already subject to various checks and balances’. On the other hand, NG resources risk to be trapped in a ‘slow-moving machine’. In order to be financed, NG-related projects need to be designed, approved and implemented as part of a process that can take several years. As a result, the timing of disbursements is just the opposite of what would be required to respond to the urgency imposed by the current situation and, even more so, by the expected collapse of incomes that the European economies are going to face.^11^ However, the Commission expects that barely 24.9% of the total new firepower for grants would be spent in 2020–2022, when the recovery needs will be greatest (Darvas 2020). Far from being a tool to counter the immediate effects of the crisis, the NG plan is more similar to the Juncker plan, and shares all its weaknesses.^12^ It is highly unlikely that countries like Italy, severely hit by the pandemic and in persistent financial distress, will be able to afford to refrain from asking for other funds (namely, ESM, SURE and others for a total amount of about 59 billion euros) which could be paid out immediately, subject to the usual conditionality.

### Long term sustainability of the EU project

The Merkel-Macron agreement has been hailed as the first step towards a more supportive Union. Behind the good intentions, there are the concrete interests of both France and Germany for the survival of the EMU: they look with growing concern at the rise of Euroscepticism in the SP. The French economy has been hit hard by the pandemic, and was already in difficulty before. GDP forecasts for 2020 vary widely, but all agree in estimating a fall in the French GDP of much the same proportions as in the case of Italy. On the other hand, Germany was, together with the Netherlands, the main beneficiary of the creation of the euro, and Italy and France were the main losers (Gasparotti and Kullas 2019).^13^ As Chancellor Merkel told the German lawmakers, “it is essential for Germany, as an export nation, that its EU partners also do well”.^14^ Indeed, the history of the EU has taught that excessive German surpluses are deleterious for the south of the Eurozone.

Greater government action, retreat from hyper-globalism, and lower growth rates predate the pandemic. The COVID-19 crisis has given yet more voice to calls for protectionist and “beggar thy neighbours” types of policies. It has led countries to prioritize resilience and autonomy in production over cost savings and efficiency through global outsourcing. The same powerful German production platform, so disproportionately export-oriented and dependent on imports of intermediate goods, finds itself vulnerable to a type of shock (the Covid-19 pandemic) that disrupts GVCs and threatens to change the existing economic order through permanent disruption of the patterns of demand and production. Although transition from an industrial platform designed for export to one for the internal market (a sort of transition from a war to a peace economy) is a formidable challenge, this transformation would benefit Germany itself, considering the winds of trade war and the growing uncertainty about the future developments of the global value chains.

## Concluding remarks

The European countries are at a crossroad between either letting the Union dissolve or radically reforming it. Today’s darkened geopolitical environment requires Europe to act as a whole. However, the EMU will remain fragile as long as it chooses to continue to delegate control over its policies to market surveillance. A true “Hamiltonian moment”, which involves adopting a common fiscal policy in support of the common monetary policy is a matter of urgency.

We still have a long way to go. Divisions between member countries marked by opposition between debtors and “frugal” creditors, as well intra-country political struggles and conflicting interests, have—even in the face of this dramatic crisis—led to the paralysis of the European institutions, with the one exception of the ECB. Faced with what she sees as a serious threat to the EU’s survival, the German Chancellor (and the Commission’s president Ursula von der Leyen) have been driven to action. However, as we argued in Sect. 3, little can be expected from the NG plan for immediate support. The ability of the SP to emerge from the crisis will increasingly depend on its ability to take advantage of the greater flexibility of EU rules for an efficient use of industrial policy, helping companies and the whole economy to respond to the challenge posed by social and technological innovation, the restructuring of production and the reorganization and shortening of GVCs.

The pandemic will have significant repercussions on the international organization of production and GVCs (on this point, see also the contributions to this Forum by Strange and Coveri et al.). Indeed, the countries initially most affected by Covid (China, Korea, Italy) are among the most important suppliers of intermediate goods at the international level. Studies on the propagation of economic shocks triggered by natural disasters (such as the earthquake that hit Japan in 2011) along the value chains (Boehm et al. 2019; Inoue and Todo 2019) found significant supplier substitution effects. Anecdotal evidence signals numerous cases of supplier substitution in some countries as a result of the coronavirus (Baldwin and Tomiura 2020). The extent of these effects depends on the degree of complexity of the production chains, which affects the degree of input substitutability. Propagation effects also depend on the presence of “hub” companies interconnected with a large number of supplier and customer firms (Inoue and Todo 2019). Future developments are uncertain, depending on the relative strength of two opposite effects. On the one hand, greater coordination afforded by digitalisation of production networks could favour substitution effects (especially in cases where value chains are less regionalised and the search for new suppliers is more difficult) (Zhenwei Quiang et al. 2020). On the other hand, processes of reshoring and shortening of value chains could occur, especially where production chains are less complex or automation is more advanced. The second possibility could represent an opportunity to reverse the processes of deindustrialization that have impoverished, above all, the productive fabric of the peripheral countries.

A third perspective, probably utopian, could contemplate coordination of coalitions of producers across EU member states. In a situation of strong productive complementarities between countries, the fortunes of the producers (workers and firms) in one country are bound to those in the other. This would call for a coordinated industrial policy at the European level aiming at ensuring a balanced development of the economies of its members through their integration in the European production networks. In emergency situations where production activities are reduced or temporarily suspended (as in the case of coronavirus shock), bilateral agreements (mediated by governments) between producers in different countries should aim at stabilizing employment levels and pre-existing supply contracts between firms through “mutualisation” of the required financial effort. After all, having surprisingly spoken out in favor of the Eurobonds, the CEO of Volkswagen Herbert Diess could—at one remove—be also supportive of such a project!

## Footnotes

1 Merkel: Germany must help other EU states get back on their feet, EURACTIV.com with Reuters 13 mag 2020 https://www.euractiv.com/section/economy-jobs/news/merkel-germany-must-help-other-eu-states-get-back-on-their-feet/.

2 These diverging trends are likely to increase as a result of the slow, small and asymmetric response that Europe is giving to the ongoing pandemic-driven economic crisis, as confirmed by the macroeconomic evidence provided in this Forum by Heimberger et al.

3 Threatening to disrupt production, the lockdown of the Italian economy following Covid-19 will make the centrality of Italian producers of specialised components for the German automotive industry clear.

4 Wage moderation played a crucial role in Spain’s automobile industry revival after the 2008 crisis. As from 2011, Spain became the top destination for FDI in the automobile industry in Europe, even outpacing the CEE countries (Pavlinek et al. 2017).

5 In 2018, when interviewed by Bild about its intention to introduce tariffs aimed at German exports, Trump was more than explicit: “When you walk down Fifth Avenue, everybody has a Mercedes-Benz parked in front of his house. How many Chevrolets do you see in Germany? Not many, maybe none”.

6 The Irish economist Emma Clancy counted 63 cases where the EU officially requested member states to cut health spending.

7 Several parties, including most recently the president of the ECB, Christine Lagarde, are urging the EC to review the Pact before its temporary suspension expires on December 31, 2020.

8 Quote reported by the website EURACTIV.com.

9 The Eurosystem balance sheet (the network of European central banks, guided by the ECB) rose from 1150 billion Euros at the beginning of 2007 to 4675 billion Euros by the end of 2018; that is, from barely 10% to almost 40% of the Euro Zone GDP (12 000 billion Euros).

10 The NG plan money will be spent over the 2021–2024 period. With an even subdivision over the period, the package amounts to an annual 0.56% of the EU's 2019 GDP, over four years.

11 It has been estimated (Fubini 2020) that in 2021 Italy could receive 4 billion euros in grants and 8 billion in loans, equal to 0.7 percent of its GDP. This compares with a slump in GDP estimated between 10 and 13 per cent in 2020. Disbursements would increase in the following years, amounting to 1.5% of GDP in 2022, 1.7% in 2023 and 2024, and 1% in 2025.

12 Art. 17.4 (a) states that “the financial contribution to be paid in instalments once the Member State has satisfactorily implemented the relevant milestones and targets identified in relation to the implementation of the recovery and resilience plan”. And art. 19.3 states that “Upon completion of the relevant agreed milestones and targets indicated in the recovery and resilience plan as approved in the implementing act of the Commission, the Member State concerned shall submit to the Commission a duly justified request for payment of the financial contribution and, where relevant, of the loan tranche”.

13 Gasparotti and Kullas estimate the additional growth of the German GDP attributable to the creation of the euro at 0.5% per annum.

14 Merkel: Germany must help other EU states get back on their feet, EURACTIV.com with Reuters 13 mag 2020 https://www.euractiv.com/section/economy-jobs/news/merkel-germany-must-help-other-eu-states-get-back-on-their-feet/

## References

[CR1] Baldwin R, Tomiura E, Baldwin R, WederDiMauro B (2020). Thinking ahead about the trade impact of Covid-19. Economics in the time of Covid-19.

[CR2] Boehm CE, Flaaen A, Pandalai-Nayar N (2019). Input linkages and the transmission of shocks: Firm-level evidence from the 2011 Tōhoku earthquake. Review of Economics and Statistics.

[CR3] Celi G, Ginzburg A, Guarascio D, Simonazzi A (2018). Crisis in the European Monetary Union. A core-periphery perspective.

[CR4] Celi G, Guarascio D, Simonazzi A (2019). Unravelling the roots of the EMU crisis. Structural divides, uneven recoveries and possible ways out. Intereconomics.

[CR5] Celi G, Ginzburg A, Guarascio D, Simonazzi A (2020). Un’unione divisiva. Una prospettiva centro-periferia della crisi europea.

[CR6] Chazan, G. (2020). Germany’s oversupply of hospital beds aids pandemic fight, The Financial Times, 14 April.

[CR7] Darvas Z. (2020). Next generation EU: 75% of grants will have to wait until 2023, Bruegel Blog, 10 June. Available at https://www.bruegel.org/2020/06/three-quarters-of-next-generation-eu-payments-will-have-to-wait-until-2023/.

[CR8] Dosi G, Guarascio D, Ricci A, Virgillito ME (2019). Neodualism in the Italian business firms: training, organizational capabilities, and productivity distributions. Small Business Economics.

[CR9] EC. (2020). Proposal for a regulation of the European Parliament and of the council establishing a recovery and resilience facility, COM (2020) 408 final 2020/0104 (COD), Brussels, May 28.

[CR10] EURACTIV.com (with Reuters) 2020. Germany gains most from relaxed EU state aid rules, 4 May. https://www.euractiv.com/section/competition/news/germany-gains-most-from-relaxed-eu-state-aid-rules/.

[CR11] Fubini, F. (2020). Fondi europei al rallentatore. Esordio con il contagocce, gli aiuti legati alle riforme. Prima tranche: 4 miliardi, 2 of June, Corriere della Sera, https://www.corriere.it/international/premium/20_giugno_01/recovery-plan-tutti-ostacoli-fondi-europei-rallentatore-prima-tranche-4-miliardi-780f3570-a447-11ea-b19d-c124828d4b5b_preview.shtml?reason=unauthenticated&cat=2&cid=1721914576&pids=PO&credits=1&origin=https%3A%2F%2Fwww.corriere.it%2Finternational%2Fpremium%2F20_giugno_01%2Frecovery-plan-tutti-ostacoli-fondi-europei-rallentatore-prima-tranche-4-miliardi-780f3570-a447-11ea-b19d-c124828d4b5b.shtml.

[CR12] Gasparotti, A., & Kullas, M. (2019). 20 Years of the Euro: Winners and losers. An empirical study, CEP Study, February. https://www.cep.eu/Studien/20_Jahre_Euro_-_Gewinner_und_Verlierer/cepStudy_20_years_Euro_-_Winners_and_Losers.pdf.

[CR13] Guarascio D, Simonazzi A (2016). A polarized country in a polarized Europe: An industrial policy for Italy’s renaissance. Economia e Politica Industriale.

[CR14] Inoue H, Todo Y (2019). Firm-level propagation of shocks through supply-chain networks. Nature Sustainability.

[CR15] Krzywdzinski M, Galgóczi B (2019). Globalisation, decarbonisation and technological change: Challenges for the German and CEE automotive supplier industry. Towards a just transition: coal, cars and the world of work.

[CR16] Pavlínek, P., Aláez-Aller, R., Gil-Canaleta, C., & Ullibarri-Arce, M. (2017). Foreign direct investment and the development of the automotive industry in Eastern and Southern Europe. ETUI Research Paper-Working Paper.

[CR17] Prante FJ, Bramucci A, Truger A (2020). Decades of tight fiscal policy have left the health care system in Italy ill-prepared to fight the COVID-19 outbreak. Intereconomics.

[CR18] Rios, B. (2020). EU countries use looser state aid rules to uphold troubled firms, EURACTIV.com. 6 Apr 2020 (updated: 21 Apr 2020) https://www.euractiv.com/section/competition/news/eu-countries-use-looser-state-aid-rules-to-uphold-troubled-firms/.

[CR19] Stehrer, R., & Stöllinger, R. (2015). The central European manufacturin core: What is driving regional production sharing? (No. 2014/15-02). FIW Research Reports.

[CR20] Tooze, A., & Schularick, M. (2020). The shock of coronavirus could split Europe—unless nations share the burden. The Guardian, 25 March.

[CR21] Walker, S., & Smith, H. (2020). Why has eastern Europe suffered less from coronavirus than the west?, The Guardian, 5 May https://www.theguardian.com/world/2020/may/05/why-has-eastern-europe-suffered-less-from-coronavirus-than-the-west.

[CR22] Wiiw. (2020). The wiiw forecasts for Central, East and Southeast Europe”, WIIW, The Vienna Institute for International Economic Studies, 6 May.

[CR23] Zhenwei Quiang, C., Li,Y., Liu, Y., Paganini, M, & Steenbergen, V. (2020). Foreign direct investment and global value chains in the wake of COVID-19: lead firms of GCV. World Bank Blog, 21 May.

